# Russian Dolls of Heme Metabolism in Malaria-Infected Red Blood Cells: Nested Vulnerabilities and Therapeutic Opportunities

**DOI:** 10.3390/pathogens15050477

**Published:** 2026-04-29

**Authors:** Swamy R. Adapa, Faiza A. Siddiqui, Rays H. Y. Jiang

**Affiliations:** 1 Department of Global, Environmental, and Genomic Health Sciences, College of Public Health, University of South Florida, Tampa, FL 33612, USA; 2 USF Genomics Program, University of South Florida, Tampa, FL 33612, USA; 3 Department of Internal Medicine, Morsani College of Medicine, University of South Florida, Tampa, FL 33612, USA

**Keywords:** malaria, heme metabolism, artemisinin, porphyrin, *Plasmodium falciparum*, heme flux, drug resistance, redox biology, host–parasite interaction

## Abstract

Heme metabolism is central to the biology of malaria parasites and to the mechanism of action of artemisinin-based therapies. Within malaria-infected red blood cells (RBCs), heme-related chemistry arises from multiple nested metabolic sources that function as “Russian dolls”: the truncated heme biosynthetic capacity of the host erythrocyte, the parasite’s own heme synthesis pathway, and host heme released through hemoglobin digestion in the parasite food vacuole. These overlapping metabolic layers create distinct pools of heme that can influence redox balance and drug activation. Recent studies highlight that exogenous 5-aminolevulinic acid (5-ALA) can perturb host heme biosynthesis in infected erythrocytes, potentially increasing intracellular levels of the heme intermediate protoporphyrin IX and sensitizing parasites to oxidative stress. However, the extent to which such metabolic perturbations affect artemisinin susceptibility depends strongly on parasite stage and exposure duration. Here we review the compartmentalized architecture of heme metabolism in malaria-infected RBCs and discuss how these nested vulnerabilities may be exploited for therapeutic intervention.

## 1. Introduction: Heme Metabolism as the Biochemical Core of Malaria Infection

Malaria remains one of the most significant infectious diseases worldwide [1], driven by the complex interplay between *Plasmodium* parasites and their host red blood cells [2]. Although mature human erythrocytes are often considered metabolically minimal due to the absence of nuclei and organelles, they represent an exceptionally heme-rich environment in which iron–porphyrin chemistry dominates cellular function [3,4]. This biochemical landscape is central to both parasite survival and the mechanism of action of frontline antimalarial therapies.

Heme plays a dual role in malaria infection and control. On one hand, it is an essential cofactor for redox reactions and oxygen transport [5,6,7]; on the other, it is a potent source of oxidative stress when released or improperly coordinated [8,9]. The parasite handles this duality through encoding a complete malarial heme biosynthesis pathway for co-factor production, digesting human hemoglobin within its food vacuole for nutrients, and detoxifying large quantities of free heme through crystallization into hemozoin [10,11]. Furthermore, heme related redox chemistry are shown to be critical for activation of artemisinin and related drugs, which rely on cleavage of their endoperoxide bridge to generate cytotoxic radicals [12]. Thus, the balance between heme utilization, sequestration, and redox activation lies at the core of malaria pathophysiology and therapeutic response.

**Figure 1:**
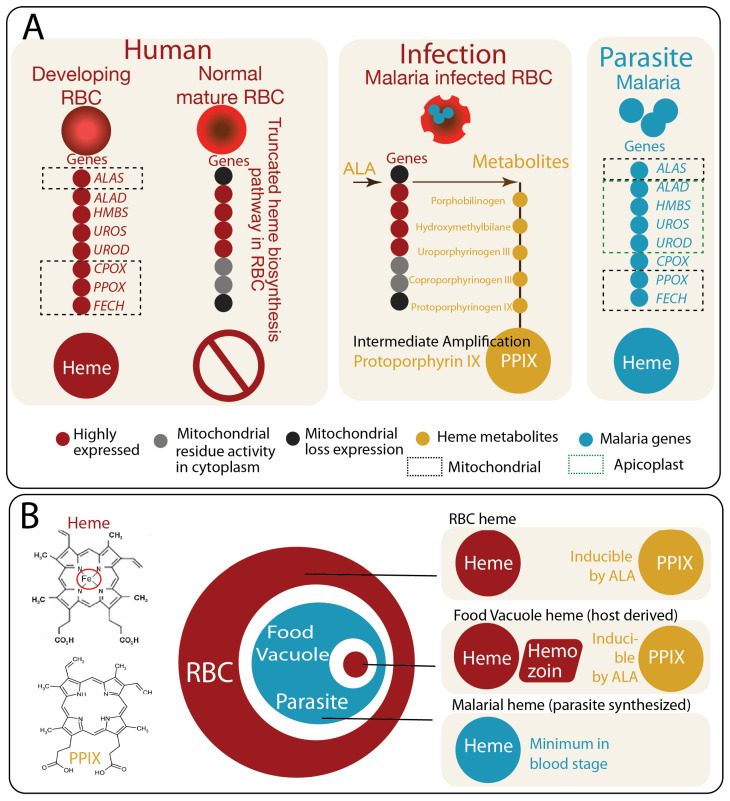
Heme biosynthesis and compartmentalized metabolite dynamics in malaria-infected erythrocytes. (**A**) Differential organization of heme biosynthesis across erythroid maturation, host cell state, and parasite metabolism. (**Left**): Heme biosynthesis exhibits distinct configurations in developing erythroid cells, mature erythrocytes, and malaria-infected erythrocytes, reflecting differences in organelle content and metabolic capacity. In immature erythroid cells, a complete heme biosynthetic pathway operates across mitochondrial and cytosolic compartments, enabling efficient conversion of precursors to PPIX and subsequent ferrochelatase-mediated insertion of iron to form heme in a high-throughput, tightly regulated process. Upon maturation, erythrocytes expel mitochondria and other organelles, resulting in a truncated pathway with minimal capacity for de novo heme synthesis. (**Middle**): Following infection by *Plasmodium* spp., mature erythrocytes undergo extensive parasite-driven remodeling, including the formation of new permeability pathways that allow uptake of small molecules such as ALA. Under these conditions, exogenous ALA can drive accumulation of upstream intermediates, particularly PPIX, selectively in infected erythrocytes. (**Right**): In parallel, the parasite retains a complete, mitochondria and apicoplast associated heme biosynthetic pathway; however, its quantitative contribution to total heme production is relatively minor compared to host-derived heme from hemoglobin digestion. (**B**) Chemical distinction and “Russian doll” compartmentalization of heme and porphyrin intermediates. The defining biochemical difference between PPIX and heme is the insertion of a central ferrous iron (Fe^2+^) into the porphyrin ring. In malaria-infected erythrocytes, heme and its precursors are distributed across nested compartments that can be conceptualized as a “Russian doll” hierarchy. (**Upper Right**): At the outer layer, the host erythrocyte contains abundant hemoglobin-derived heme. Upon ALA exposure, the truncated host pathway in infected erythrocytes can be re-engaged to produce and accumulate PPIX. (**Middle Right**): Within the parasite, hemoglobin-derived heme is released in the food vacuole and detoxified through crystallization into hemozoin. This compartment may also interface with host-derived intermediates under conditions of increased precursor availability. (**Lower Right**): At the innermost layer, the parasite maintains its own endogenous heme biosynthetic pathway, contributing a smaller but functionally distinct pool of heme. Abbreviations: PPIX, protoporphyrin IX; ALA, 5-aminolevulinic acid; ALAS, 5-aminolevulinate synthase; ALAD, 5-aminolevulinate dehydratase; HMBS, hydroxymethylbilane synthase; UROS, uroporphyrinogen III synthase; UROD, uroporphyrinogen decarboxylase; CPOX, coproporphyrinogen oxidase; PPOX, protoporphyrinogen oxidase; FECH, ferrochelatase.

A defining but underappreciated feature of malaria-infected RBCs is the presence of multiple, spatially and functionally distinct heme-related compartments [2,13,14,15] (Figure 1A). These include the residual metabolic capacity of the host erythrocyte [15], the parasite’s own heme biosynthetic machinery [16], and the food vacuole where hemoglobin degradation occurs [17]. Each of these layers contributes to distinct pools of heme and inducible porphyrin intermediates, which differ in chemical state, localization, and biological function. Importantly, parasite-induced permeability changes allow exogenous metabolites such as ALA, the first committed heme precursor (Figure 1A), to enter the host cell [14,15], enabling experimental and potentially therapeutic manipulation of porphyrin metabolism within this system.

We propose that artemisinin susceptibility is not determined by total heme abundance, but by the distinction between bound and labile heme, as well as between heme and porphyrin intermediates within infected erythrocytes. Failure to distinguish these chemically distinct pools can lead to misinterpretation of heme-dependent drug responses.

In this review, we conceptualize these interconnected layers as “Russian dolls of heme metabolism”, in which nested metabolic compartments generate overlapping but non-equivalent pools of heme and its precursors (Figure 1B). This framework highlights how host and parasite pathways are integrated yet differentially regulated, creating unique vulnerabilities that can be exploited therapeutically. By distinguishing between bound and labile heme, and between heme and porphyrin intermediates such as protoporphyrin IX, we aim to provide a unified view of how heme metabolism shapes parasite biology, drug activation, and redox balance within infected erythrocytes.

## 2. Heme Versus Porphyrin: Chemical Foundations of Redox Reactivity

A clear distinction between heme and porphyrin intermediates is essential for understanding the biochemical basis of malaria pathophysiology and drug action, yet these terms are often used interchangeably in the literature. Chemically, heme refers to iron-coordinated protoporphyrin IX (Fe–PPIX), in which a central ferrous iron (Fe^2+^) is inserted into the tetrapyrrole macrocycle by ferrochelatase [6]. In contrast, porphyrins such as protoporphyrin IX (PPIX) lack a coordinated metal center [5,18,19] and therefore exhibit distinct physicochemical and redox properties.

**Figure 2:**
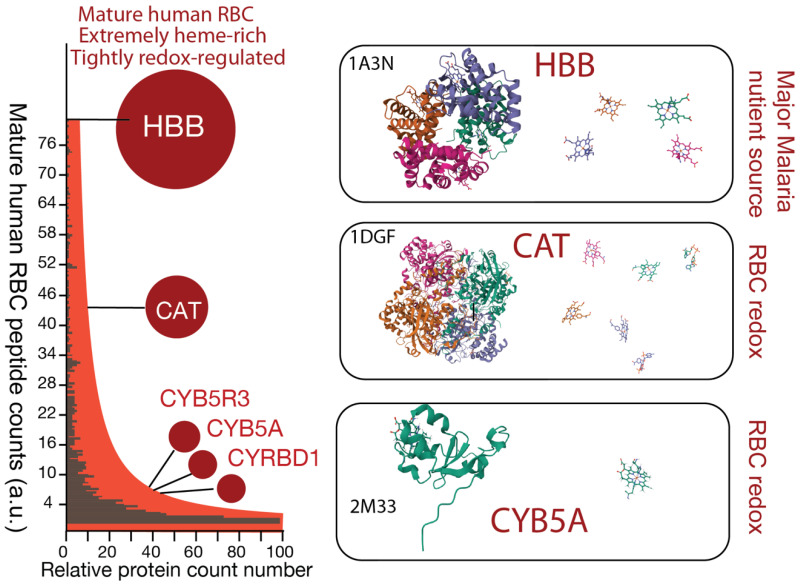
Hemoprotein abundance and structural organization in the mature human erythrocyte. Proteomic analyses of mature human red blood cells reveal an extremely heme-rich proteome dominated by tightly regulated hemoproteins involved in oxygen transport and redox homeostasis. (**Left**): Quantitative proteomic profiling based on peptide abundance demonstrates a high enrichment of hemoproteins. Hemoglobin subunit beta (HBB), the most abundant protein in RBCs, is estimated at ~10^9^ molecules per cell and was excluded from scale for visualization purposes. Among the remaining proteins, catalase (CAT) and redox-associated enzymes, including cytochrome b5 reductase 3 (CYB5R3), cytochrome b5 type A (CYB5A), and cytochrome b reductase domain-containing protein 1 (CYBRD1), are present at relatively high abundance, reflecting the strong redox buffering capacity of mature RBCs. Mature RBC proteome data retrieved from Bryk et al. [20]. The y-axis represents peptide counts (arbitrary units), and the x-axis indicates relative protein abundance. (**Right**): Representative three-dimensional structures of major hemoproteins in mature human RBCs. Hemoglobin (HBB; PDB: 1A3N), the primary oxygen carrier and a major nutrient source for *Plasmodium*, contains four heme groups per tetramer. Catalase (CAT; PDB: 1DGF), a key regulator of oxidative stress throughout the RBC lifespan, also contains four heme groups per functional unit. Cytochrome b5 (CYB5A; PDB: 2M33) and related redox proteins are present at lower but significant abundance, each binding a single heme molecule. Notably, nearly all hemoproteins in mature RBCs are synthesized during erythroid development prior to organelle loss. Consequently, heme in mature RBCs exists almost entirely in a protein-bound state, with minimal to no detectable labile heme pool.

This difference in metal coordination fundamentally alters reactivity. Proteomic studies [20] of mature RBCs have shown an extremely heme-rich proteome dominated by hemoproteins (Figure 2). Heme (Fe–PPIX), when tightly bound within RBC proteins such as hemoglobin or cytochromes, as illustrated in Figure 2, is relatively stable and functions as a controlled redox cofactor, enabling electron transfer and catalytic reactions [21]. However, when released from protein coordination, as occurs during hemoglobin digestion in *Plasmodium*, heme becomes potentially labile and highly reactive. Labile heme can catalyze the formation of reactive oxygen species (ROS) through Fenton-like chemistry, contributing to oxidative stress and cellular damage [8,22,23]. To mitigate this toxicity, malaria parasites rapidly convert heme into inert crystalline hemozoin within the food vacuole [10,11,17,24].

In contrast, metal-free porphyrins, i.e., heme intermediates, such as PPIX possess strong photodynamic and redox-amplifying properties [8]. The conjugated ring structure of porphyrins enables efficient absorption of light and energy transfer, leading to the generation of singlet oxygen and other reactive species [8]. Even in the absence of light, porphyrins can participate in redox cycling and sensitize cells to oxidative stress [22]. These properties make porphyrins potent amplifiers of oxidative damage, particularly under conditions of metabolic imbalance or elevated precursor flux.

In this context, PPIX can be considered a “forbidden” intermediate in normal physiology [25,26,27,28], as its high chemical reactivity and lack of a defined functional role necessitate rapid conversion to heme to prevent accumulation.

Importantly, the balance between heme and porphyrin intermediates is tightly regulated under physiological conditions. In developing erythroid cells, rapid and coordinated flux through the heme biosynthetic pathway ensures efficient conversion of PPIX to heme, preventing accumulation of toxic intermediates such as PPIX [7,21,29,30]. However, in mature erythrocytes and especially in malaria-infected cells, this balance can be perturbed. Increased availability of precursors such as ALA, combined with altered cellular permeability and metabolic remodeling, can lead to accumulation of PPIX within infected RBCs [14,15].

This distinction has direct implications for antimalarial therapy. Artemisinin and its derivatives have been shown to be activated by cleavage of their endoperoxide bridge, a process facilitated by heme [12]. In parallel, accumulation of porphyrin intermediates such as PPIX have been proposed to enhance oxidative stress and potentially sensitize parasites to drug-induced damage [14]. Although not yet directly demonstrated in malaria, studies in other systems suggest that porphyrin-mediated redox amplification occurs in diffuse, lipid-associated microenvironments, where intermediates can support propagation of reactive species [22,31]. Thus, heme and porphyrins represent chemically distinct but functionally interconnected mediators of redox biology in malaria, with complementary roles in parasite survival and therapeutic vulnerability.

## 3. Bound Heme Versus Labile Heme Pools: Control of Redox Activity and Drug Activation

A central concept in heme biology is the distinction between protein-bound heme and labile heme pools, which differ fundamentally in chemical reactivity, cellular localization, and biological function. In mature human erythrocytes, the overwhelming majority of heme exists in a tightly coordinated, protein-bound state, primarily within hemoglobin [32]. This bound heme is structurally constrained, redox-regulated, and largely inert with respect to uncontrolled chemical reactivity, enabling efficient oxygen transport while minimizing oxidative damage. Additional hemoproteins present in mature human red blood cells, including catalase and cytochrome b5 systems, further contribute to redox buffering [3,4], maintaining a highly controlled intracellular environment (Figure 2).

In contrast, labile heme refers to a transient, low-abundance pool of heme that is not permanently coordinated within proteins [33,34]. This pool is chemically reactive and capable of participating in redox cycling, iron release, and radical generation. Although small in quantity, labile heme plays a disproportionate role in cellular signaling and oxidative stress. Its generation is tightly regulated under physiological conditions to prevent toxicity.

It should be noted that, in contrast to the intermediate PPIX, which lacks a defined physiological function, labile heme, despite its low abundance and reactivity, serves regulated roles in cellular signaling and redox sensing [35,36,37].

Malaria infection fundamentally perturbs this balance. During the intraerythrocytic stage, *Plasmodium* parasites digest host hemoglobin within the acidic food vacuole, releasing large amounts of heme [17]. This process creates a localized surge in labile heme, which, if not rapidly detoxified, can induce lethal oxidative stress. To survive, the parasite converts heme into inert crystalline hemozoin, effectively sequestering it and limiting its redox activity. However, this detoxification process is not instantaneous, and transient exposure to labile heme creates a window of vulnerability.

This labile heme pool is proposed to be central to the mechanism of action of artemisinin and related endoperoxide antimalarials. Activation of artemisinin requires cleavage of its endoperoxide bridge, a reaction facilitated by heme or heme-derived species. The resulting carbon-centered radicals can alkylate proteins, lipids, and other macromolecules, leading to widespread cellular damage and parasite death. Thus, the efficacy of artemisinin is closely linked to the dynamics of labile heme generation and detoxification within the parasite [12].

This spatial and biochemical heterogeneity creates distinct redox microenvironments within the infected cell. Understanding the balance between bound and labile heme is therefore critical for defining both parasite survival strategies and therapeutic vulnerabilities. While bound heme represents a stable reservoir, labile heme constitutes a dynamic and reactive pool that drives oxidative stress and drug activation.

## 4. The Three Russian Dolls of Heme Metabolism: Nested Compartments and Functional Heterogeneity

A defining feature of malaria-infected red blood cells is the coexistence of multiple, spatially nested heme metabolic systems that differ in origin, capacity, and biochemical function. These systems can be conceptualized as “Russian dolls of heme metabolism,” in which host and parasite pathways are layered within one another, generating distinct but interacting pools of heme and porphyrin intermediates. This compartmentalization is not merely structural; it creates functional heterogeneity in redox activity, metabolic flux, and drug susceptibility.

### 4.1. Host Erythrocyte Heme Metabolism: A Truncated but Inducible System

Mature human RBCs lack nuclei and mitochondria and are therefore incapable of sustaining a complete heme biosynthetic pathway. During erythroid development, however, these cells actively synthesize large quantities of heme through coordinated mitochondrial and cytosolic enzymatic steps, resulting in a proteome that is exceptionally rich in hemoproteins with bound-heme, particularly hemoglobin [4,20]. Upon maturation, organelle loss truncates this pathway, leaving the mature RBC with minimal capacity for de novo heme synthesis [14] (Figure 1A).

Despite this limitation, the host erythrocyte retains residual enzymatic components for heme biosynthesis. Under normal physiological conditions, flux through the heme pathway is negligible, and porphyrin intermediates do not accumulate [14,15]. However, in malaria-infected RBCs, parasite-induced remodeling increases membrane permeability, allowing uptake of small metabolites such as ALA [14,15]. Under these conditions, the truncated host pathway can be partially re-engaged, leading to accumulation of upstream intermediates such as PPIX. This inducible capacity represents a latent metabolic layer that is normally silent but can be experimentally or therapeutically exploited.

### 4.2. Parasite Heme Biosynthesis: A Complete but Quantitatively Minor Pathway

In contrast to the host RBC, *Plasmodium* parasites retain a complete heme biosynthetic pathway distributed across the mitochondrion and apicoplast [15,16] (Figure 1A). This pathway includes all canonical enzymatic steps required for the synthesis of heme from precursor metabolites. However, the functional importance of parasite-derived heme remains a subject of debate. Genetic and biochemical studies suggest that while the pathway is intact, its quantitative contribution to the parasite’s total heme pool is relatively minor compared to heme obtained from host hemoglobin digestion. Stable isotope tracing studies demonstrate that de novo heme synthesis contributes minimally to blood-stage parasites, which primarily rely on host hemoglobin digestion as a dominant heme source [16].

Nevertheless, parasite heme biosynthesis may play specialized roles, particularly in mitochondrial function and electron transport in sexual stages of the parasite development [15,16]. The spatial separation of this pathway from the food vacuole and host cytoplasm further distinguishes it as a discrete metabolic compartment. As such, it represents a potential target for intervention, especially in contexts where reliance on endogenous heme synthesis may increase, such as sexual developmental stages or under drug pressure.

### 4.3. Parasite Food Vacuole: Host-Derived Heme and Heme Metabolites

The most significant source of parasite acquired heme in malaria-infected RBCs arises from the digestion of host hemoglobin within the parasite food vacuole. During this process, proteolytic degradation releases large quantities of heme, creating a localized environment enriched in labile, redox-active species. This pool represents both a critical nutrient source [16] and a major toxic threat to the parasite.

To mitigate heme toxicity, the parasite rapidly converts labile heme into inert crystalline hemozoin, sequestering iron and limiting uncontrolled redox reactions. Notably, this dynamic balance is closely linked to artemisinin susceptibility and resistance [38,39,40,41,42]. Mutations in the Kelch13 (K13) pathway are associated with reduced hemoglobin uptake and delayed digestion [38,41,42]. Within the “Russian doll” framework, these K13 alterations have three related implications in drug resistance: (i) reduced trafficking of host-derived hemoglobin from the outer host compartment to the food vacuole, (ii) altered transfer of digestion products and host-derived nutrients from the food vacuole to parasite compartments, and (iii) diminished propagation of redox stress generated in the food vacuole into the parasite’s intracellular biochemical networks. In this context, the food vacuole serves not only as an infection hub of heme metabolism, but also as a regulatory node in which modulation of heme release and detoxification reshapes redox vulnerability and contributes to resistance phenotypes [42].

## 5. Targeting Heme: Artemisinin and Related Endoperoxide Antimalarials

Artemisinin and its derivatives, including dihydroartemisinin (DHA), artesunate, and artemether, remain the cornerstone of modern antimalarial therapy. Their rapid parasiticidal activity depends on heme chemistry, exploiting the heme-rich environment generated by hemoglobin digestion within malaria-infected red blood cells.

Parasite stage plays a critical role in determining susceptibility to artemisinin [38,39,42,43]. Early ring-stage parasites exhibit reduced sensitivity, which has been linked to lower rates of hemoglobin digestion and consequently reduced generation of labile heme. In contrast, trophozoite-stage parasites, characterized by active hemoglobin catabolism, are highly susceptible. This stage-dependent variation underscores the importance of heme flux rather than total heme abundance in governing drug activation.

A critical but often underappreciated variable is time. Heme metabolism within infected erythrocytes evolves across the intraerythrocytic cycle, with early ring stages characterized by low hemoglobin digestion and limited flux, and trophozoite stages exhibiting high heme turnover and increased metabolic activity. As a result, short-duration measurements capture fundamentally different biochemical states than sustained perturbations.

Within the “Russian doll” framework, resistance can be interpreted as a reprogramming of heme metabolism that minimizes exposure to labile heme in the food vacuole compartment, thereby reducing the effective activation of the drug.

Importantly, artemisinin does not act through a single molecular target but rather induces widespread damage through radical-mediated alkylation [9,12]. This broad mechanism is both a strength, in terms of potency, and a vulnerability, as it depends on the presence of activating chemical species. Strategies that increase labile heme availability or enhance redox stress, such as perturbation of heme detoxification or induction of porphyrin accumulation, have the potential to potentiate artemisinin activity.

Recent work also highlights that the relationship between heme and artemisinin activity is not strictly monotonic. Zhu and Zhou (2022) [44] systematically increased intracellular free heme through multiple approaches, including modulation of heme synthesis, hemoglobin digestion, and use of heme analogs, and consistently observed a reduction in artemisinin potency. Restoration of drug activity upon lowering labile heme levels suggests that excessive labile heme can act as a sink or buffer for reactive intermediates, dampening effective radical propagation.

These findings point to a tightly regulated heme homeostasis network within the infected RBC, in which both insufficient and excessive labile heme can limit drug efficacy. Within this context, targeting heme requires careful consideration of dose, timing, and compartmentalization, as perturbations that simply elevate total heme may paradoxically reduce, rather than enhance, artemisinin activity.

Taken together, heme-mediated activation of artemisinin can be self-limiting. The iron center and associated redox networks can both generate and constrain radical intermediates, for example, through redox cycling and interactions with cellular antioxidants, such that a portion of the drug may be consumed without sustained propagation of damage [44]. In contrast, PPIX, lacking a metal center and integration into buffering pathways, permits radical propagation and amplifies oxidative stress, consistent with its well-established role as a ROS-generating and cytotoxic intermediate in photodynamic systems [45,46,47].

## 6. Targeting Porphyrin Metabolism: Inducing Intermediate Accumulation as a Therapeutic Strategy

### 6.1. Reactivating Host Porphyrin Metabolism in Infected Erythrocytes

While most antimalarial strategies focus on disrupting heme utilization or detoxification [38,39,44], an alternative and less explored approach is to perturb upstream porphyrin metabolism to induce accumulation of reactive intermediates. In the context of malaria-infected RBCs, this strategy leverages the unique metabolic architecture described in the “Russian doll” framework, in which otherwise silent or tightly controlled pathways can be reactivated or overloaded to generate cytotoxic stress.

A central molecule in this approach is 5-ALA, the first committed precursor in the heme biosynthetic pathway. Under normal physiological conditions, mature RBCs lack the organelles required for sustained heme synthesis and therefore exhibit minimal flux through this pathway. However, infection by Plasmodium induces profound remodeling of the host cell, including the formation of new permeability pathways that allow uptake of exogenous metabolites such as ALA [14,15]. This altered permeability could enable experimental manipulation of elevating porphyrin production within infected RBCs.

Upon uptake in infected RBCs, ALA can be metabolized through the residual host enzymatic machinery to produce PPIX. Unlike heme, which is typically protein-bound and regulated, PPIX is a metal-free porphyrin with strong redox and photodynamic properties. PPIX accumulation represents a shift from a controlled metabolic state to one characterized by intermediate buildup and increased chemical reactivity [22].

### 6.2. PPIX as a Photodynamic and Redox Amplifier of Parasite Vulnerability

The cytotoxic potential of PPIX arises from its ability to amplify oxidative stress. The extended conjugated ring structure of porphyrins facilitates electron transfer and can promote the generation of reactive oxygen species (ROS) [22] through photodynamic processes [22] in the presence of light. Even in non-illuminated conditions, elevated levels of porphyrins can sensitize cells to oxidative damage [45].

Importantly, induction of porphyrin accumulation does not merely add an independent source of oxidative stress but can intersect with existing heme-dependent pathways. Porphyrin accumulation may enhance radical propagation in multiple compartments [22,27], as lipid-based radicals have been shown to diffuse and propagate in other cellular systems [46]. These radical reactions suggest that porphyrin metabolism can act synergistically with endoperoxide drugs, such as artemisinin, to amplify parasite killing by reaching across compartments.

Experimental studies have demonstrated that ALA-induced porphyrin accumulation can selectively occur in infected erythrocytes and can increase susceptibility of parasites to oxidative stress. However, the magnitude and duration of this effect are highly dependent on parasite developmental stage, exposure conditions, and the capacity of both host and parasite systems to buffer intermediate accumulation.

### 6.3. Time- and Flux-Dependent Sensitization Across the Intraerythrocytic Cycle

From a therapeutic perspective, targeting porphyrin metabolism represents a strategy of “intermediate amplification,” in which metabolic flux is deliberately driven toward accumulation of reactive intermediates rather than completion of the pathway. This approach contrasts with classical enzyme inhibition and instead exploits the inherent instability of partially completed biosynthetic processes.

Within the “Russian doll” model, induction of porphyrin accumulation primarily affects the outer host-derived layer but can propagate inward, influencing food vacuole and parasite compartments. This cross-compartmental effect underscores the interconnected nature of heme metabolism in infected RBCs and suggests that interventions targeting one layer can have system-wide consequences.

A key distinction is that 5-ALA–mediated sensitization operates through host-derived porphyrin metabolism [14], rather than direct modulation of parasite heme pools [48]. In mature erythrocytes, the truncated heme biosynthetic pathway is inefficient at completing iron insertion and is normally minimally active. During infection, iRBCs become more permeable, allowing exogenous 5-ALA to enter and drive accumulation of upstream intermediates, particularly PPIX. Unlike protein-bound heme, PPIX is chemically unconstrained and can amplify redox reactions, acting as a reservoir of latent oxidative potential. This host-derived PPIX pool is therefore mechanistically distinct from the transient labile heme generated during hemoglobin digestion in the parasite food vacuole.

Critically, this process is time- and flux-dependent [14,48]. During the 6-h Ring-Stage survival Assay (RSA) A window (0–6 h post-invasion), ALA uptake and porphyrin accumulation are limited, and short-term co-exposure with DHA (6–9 h) may not substantially alter redox balance. However, when ALA is maintained following DHA washout and sustained over 24–72 h, spanning the transition from ring to trophozoite stages, porphyrin intermediates progressively accumulate within infected erythrocytes. This extended exposure window coincides with increased metabolic activity and permeability, enabling amplification of oxidative stress and enhancing DHA-induced damage. Thus, targeting PPIX reflects a cumulative metabolic sensitization mechanism, which emerges over a full intraerythrocytic cycle rather than within the acute RSA timeframe.

### 6.4. Reconciling Conflicting Observations in ALA–Artemisinin Studies

Apparent discrepancies between studies examining ALA–artemisinin interactions may reflect differences in experimental regime rather than true contradiction [14,48]. Short-timescale assays focused on early ring stages (0–6 h) primarily capture low-flux metabolic states with limited porphyrin accumulation, whereas longer exposures (24–72 h) integrate across the intraerythrocytic cycle, allowing progressive buildup of intermediates such as PPIX and amplification of oxidative stress across compartments. Within this framework, existing datasets can be interpreted as sampling different temporal and metabolic windows of the same underlying system, rather than representing mutually exclusive outcomes.

## 7. Future Directions: Exploiting Host Erythrocyte Biology and Nested Metabolic Vulnerabilities

The “Russian doll” framework reframes malaria as a systems-level interaction between host erythrocyte biology and parasite metabolism, opening opportunities beyond parasite-centric targeting. Infection-induced permeability creates a selective gateway into infected RBCs, enabling delivery of metabolic modulators and redox-active compounds that may bypass classical resistance mechanisms.

Food vacuole physiology, including pH-dependent heme crystallization [49,50,51], may regulate compartmental heme availability. Additionally, transport processes at the parasite food vacuole, including those mediated by the *P. falciparum* chloroquine resistance transporter (PfCRT), are known to regulate solute flux and drug access [52,53,54] and may influence heme or porphyrin compartmentalization, although direct evidence for effects on PPIX dynamics remains limited. Future isotope-based studies may help resolve the compartmental distribution of PPIX.

Human genetic variation further underscores the importance of host context. Hemoglobinopathies reshape erythrocyte redox balance and heme handling [3,55,56,57], suggesting that endogenous host protective states may be pharmacologically mimicked.

Targeting porphyrin and heme dynamics offers a complementary strategy: driving accumulation of reactive intermediates or disrupting detoxification can shift the system toward oxidative stress unfavorable for parasite survival. Such approaches are likely most effective when combined across compartments, for example, pairing porphyrin induction in the host with inhibition of heme detoxification in the parasite.

Advances in metabolomics, imaging, and single-cell approaches will be essential to resolve heme flux and spatial heterogeneity across parasite stages [58,59], enabling precise targeting of these vulnerabilities.

More broadly, nested metabolic organization may represent a general principle of host–pathogen systems. In malaria, this architecture reveals a central insight: heme metabolism in malaria is not a static resource, but a dynamic, compartmentalized system whose imbalance, rather than abundance, defines vulnerability.

## Data Availability

All relevant data are contained within the manuscript.

## References

[1] Zhang M., Wang C., Otto T.D., Oberstaller J., Liao X., Adapa S.R., Udenze K., Bronner I.F., Casandra D., Mayho M. (2018). Uncovering the essential genes of the human malaria parasite Plasmodium falciparum by saturation mutagenesis. Science.

[2] Mohandas N., An X. (2012). Malaria and human red blood cells. Med. Microbiol. Immunol..

[3] Möller M.N., Orrico F., Villar S.F., López A.C., Silva N., Donzé M., Thomson L., Denicola A. (2023). Oxidants and Antioxidants in the Redox Biochemistry of Human Red Blood Cells. ACS Omega.

[4] Sae-Lee W., McCafferty C.L., Verbeke E.J., Havugimana P.C., Papoulas O., McWhite C.D., Houser J.R., Vanuytsel K., Murphy G.J., Drew K. (2022). The protein organization of a red blood cell. Cell Rep..

[5] Fratz E.J., Hunter G.A., Ferreira G.C. (2014). Expression of Murine 5-Aminolevulinate Synthase Variants Causes Protoporphyrin IX Accumulation and Light-Induced Mammalian Cell Death. PLoS ONE.

[6] Shi Z., Ferreira G.C. (2003). A continuous anaerobic fluorimetric assay for ferrochelatase by monitoring porphyrin disappearance. Anal. Biochem..

[7] Zhang J., Ferreira G.C. (2002). Transient State Kinetic Investigation of 5-Aminolevulinate Synthase Reaction Mechanism. J. Biol. Chem..

[8] Pignatelli P., Umme S., D’Antonio D.L., Piattelli A., Curia M.C. (2023). Reactive Oxygen Species Produced by 5-Aminolevulinic Acid Photodynamic Therapy in the Treatment of Cancer. Int. J. Mol. Sci..

[9] Taubenschmid-Stowers J., Orthofer M., Laemmerer A., Krauditsch C., Rózsová M., Studer C., Lötsch D., Gojo J., Gabler L., Dyczynski M. (2023). A whole-genome scan for Artemisinin cytotoxicity reveals a novel therapy for human brain tumors. EMBO Mol. Med..

[10] Weissbuch I., Leiserowitz L. (2008). Interplay between malaria; crystalline hemozoin formation, and antimalarial drug action and design. Chem. Rev..

[11] Egan T.J. (2002). Physico-chemical aspects of hemozoin (malaria pigment) structure and formation. J. Inorg. Biochem..

[12] Wang J., Zhang C.-J., Ni Chia W., Loh C.C.Y., Li Z., Lee Y.M., He Y., Yuan L.-X., Lim T.K., Liu M. (2015). Haem-activated promiscuous targeting of artemisinin in Plasmodium falciparum. Nat. Commun..

[13] Cooke B.M., Mohandas N., Coppel R.L. (2001). The malaria-infected red blood cell: Structural and functional changes. Adv. Parasitol..

[14] Siddiqui F.A., Adapa S.R., Li X., Miao J., Cui L., Jiang R.H.Y. (2026). Targeting Infected Host Cell Heme Metabolism to Kill Malaria Parasites. Pharmaceuticals.

[15] Sigala P.A., Crowley J.R., Henderson J.P., Goldberg D.E. (2015). Deconvoluting heme biosynthesis to target blood-stage malaria parasites. eLife.

[16] Nagaraj V.A., Sundaram B., Varadarajan N.M., Subramani P.A., Kalappa D.M., Ghosh S.K., Padmanaban G. (2013). Malaria parasite-synthesized heme is essential in the mosquito and liver stages and complements host heme in the blood stages of infection. PLoS Pathog..

[17] Banerjee R., Goldberg D.E. (2001). Antimalarial Chemotherapy: Mechanisms of Action, Resistance, and New Directions in Drug Discovery.

[18] Gillam M.E., Hunter G.A., Ferreira G.C. (2018). Ferrochelatase π-helix: Implications from examining the role of the conserved π-helix glutamates in porphyrin metalation and product release. Arch. Biochem. Biophys..

[19] Hunter G.A., Vankayala S.L., Gillam M.E., Kearns F.L., Woodcock H.L., Ferreira G.C. (2016). The conserved active site histidine-glutamate pair of ferrochelatase coordinately catalyzes porphyrin metalation. J. Porphyr. Phthalocyanines.

[20] Bryk A.H., Wiśniewski J.R. (2017). Quantitative Analysis of Human Red Blood Cell Proteome. J. Proteome Res..

[21] Ferreira G.C. (1995). Heme biosynthesis: Biochemistry; molecular biology, and relationship to disease. J. Bioenerg. Biomembr..

[22] Lynch J., Wang Y., Li Y., Kavdia K., Fukuda Y., Ranjit S., Robinson C.G., Grace C.R., Xia Y., Peng J. (2023). A PPIX-binding probe facilitates discovery of PPIX-induced cell death modulation by peroxiredoxin. Commun. Biol..

[23] Stölzel U., Kubisch I., Stauch T., Schuppan D. (2022). Physician’s Guide to the Diagnosis, Treatment, and Follow-Up of Inherited Metabolic Diseases.

[24] Egan T.J. (2008). Recent advances in understanding the mechanism of hemozoin (malaria pigment) formation. J. Inorg. Biochem..

[25] Adapa S.R., Sami A., Meshram P., Ferreira G.C., Jiang R.H. (2024). Uncovering Porphyrin Accumulation in the Tumor Microenvironment. Genes.

[26] Adapa S.R., Porshe S., Talada D.P., Nywening T.M., Anderson M.L., Shaw T.I., Jiang R.H.Y. (2025). Spatial Transcriptomics Reveals Distinct Architectures but Shared Vulnerabilities in Primary and Metastatic Liver Tumors. Cancers.

[27] Adapa S.R., Meshram P., Sami A., Jiang R.H. (2024). Harnessing Porphyrin Accumulation in Liver Cancer: Combining Genomic Data and Drug Targeting. Biomolecules.

[28] Adapa S.R., A Hunter G., E Amin N., Marinescu C., Borsky A., Sagatys E.M., Sebti S.M., Reuther G.W., Ferreira G.C., Jiang R.H. (2024). Porphyrin overdrive rewires cancer cell metabolism. Life Sci. Alliance.

[29] Corrons J.L.V., Casafont L.B., Frasnedo E.F. (2021). Concise review: How do red blood cells born, live, and die?. Ann. Hematol..

[30] Stojanovski B.M., Hunter G.A., Na I., Uversky V.N., Jiang R.H., Ferreira G.C. (2019). 5-Aminolevulinate synthase catalysis: The catcher in heme biosynthesis. Mol. Genet. Metab..

[31] Xu H., Sun Y., Zhang Y., Wang W., Dan J., Yao J., Chen H., Tian F., Sun X., Guo S. (2014). Protoporphyrin IX induces a necrotic cell death in human THP-1 macrophages through activation of reactive oxygen species/c-Jun N-terminal protein kinase pathway and opening of mitochondrial permeability transition pore. Cell. Physiol. Biochem..

[32] D’alessandro A., Anastasiadi A.T., Tzounakas V.L., Nemkov T., Reisz J.A., Kriebardis A.G., Zimring J.C., Spitalnik S.L., Busch M.P. (2023). Red Blood Cell Metabolism In Vivo and In Vitro. Metabolites.

[33] Gouveia Z., Carlos A.R., Yuan X., Aires-Da-Silva F., Stocker R., Maghzal G.J., Leal S.S., Gomes C.M., Todorovic S., Iranzo O. (2017). Characterization of plasma labile heme in hemolytic conditions. FEBS J..

[34] Soares M.P., Bozza M.T. (2016). Red alert: Labile heme is an alarmin. Curr. Opin. Immunol..

[35] Shimizu T., Lengalova A., Martínek V., Martínková M. (2019). Heme: Emergent roles of heme in signal transduction, functional regulation and as catalytic centres. Chem. Soc. Rev..

[36] Comer J.M., Zhang L. (2018). Experimental methods for studying cellular heme signaling. Cells.

[37] Hanna D.A., Martinez-Guzman O., Reddi A.R. (2017). Heme gazing: Illuminating eukaryotic heme trafficking, dynamics, and signaling with fluorescent heme sensors. Biochemistry.

[38] Birnbaum J., Scharf S., Schmidt S., Jonscher E., Hoeijmakers W.A.M., Flemming S., Toenhake C.G., Schmitt M., Sabitzki R., Bergmann B. (2020). A Kelch13-defined endocytosis pathway mediates artemisinin resistance in malaria parasites. Science.

[39] Gibbons J., Button-Simons K.A., Adapa S.R., Li S., Pietsch M., Zhang M., Liao X., Adams J.H., Ferdig M.T., Jiang R.H.Y. (2018). Altered expression of K13 disrupts DNA replication and repair in Plasmodium falciparum. BMC Genom..

[40] Hunt P., Afonso A., Creasey A., Culleton R., Sidhu A.B.S., Logan J., Valderramos S.G., McNae I., Cheesman S., Rosario V.D. (2007). Gene encoding a deubiquitinating enzyme is mutated in artesunate- and chloroquine-resistant rodent malaria parasites. Mol. Microbiol..

[41] Ménard D., Khim N., Beghain J., Adegnika A.A., Shafiul-Alam M., Amodu O., Rahim-Awab G., Barnadas C., Berry A., Boum Y. (2016). A Worldwide Map of Plasmodium falciparum K13-Propeller Polymorphisms. N. Engl. J. Med..

[42] Siddiqui F.A., Liang X., Cui L. (2021). Plasmodium falciparum resistance to ACTs: Emergence, mechanisms, and outlook. Int. J. Parasitol. Drugs Drug Resist..

[43] Simmons C.F., Gibbons J., Zhang M., Oberstaller J., Pires C.V., Casandra D., Wang C., Seyfang A., Otto T.D., Rayner J.C. (2023). Protein KIC5 is a novel regulator of artemisinin stress response in the malaria parasite Plasmodium falciparum. Sci. Rep..

[44] Zhu P., Zhou B. (2022). The Antagonizing Role of Heme in the Antimalarial Function of Artemisinin: Elevating Intracellular Free Heme Negatively Impacts Artemisinin Activity in Plasmodium falciparum. Molecules.

[45] Takahashi J., Misawa M. (2009). Characterization of reactive oxygen species generated by protoporphyrin IX under X-ray irradiation. Radiat. Phys. Chem..

[46] Sachar M., Anderson K.E., Ma X. (2016). Protoporphyrin IX: The Good, the Bad, and the Ugly. J. Pharmacol. Exp. Ther..

[47] Wyld L., Burn J., Reed M., Brown N. (1997). Factors affecting aminolaevulinic acid-induced generation of protoporphyrin IX. Br. J. Cancer.

[48] Behrens H.M., Henshall I.G., Spielmann T. (2026). 5-ALA does not potentiate dihydroartemisinin against Plasmodium falciparum malaria parasites. EMBO Mol. Med..

[49] Rush M.A., Baniecki M.L., Mazitschek R., Cortese J.F., Wiegand R., Clardy J., Wirth D.F. (2009). Colorimetric High-Throughput Screen for Detection of Heme Crystallization Inhibitors. Antimicrob. Agents Chemother..

[50] Hoang A.N., Sandlin R.D., Omar A., Egan T.J., Wright D.W. (2010). The neutral lipid composition present in the digestive vacuole of Plasmodium falciparum concentrates heme and mediates β-hematin formation with an unusually low activation energy. Biochemistry.

[51] Coronado L.M., Nadovich C.T., Spadafora C. (2014). Malarial hemozoin: From target to tool. Biochim. Biophys. Acta.

[52] Martin R.E., Marchetti R.V., Cowan A.I., Howitt S.M., Bröer S., Kirk K. (2009). Chloroquine Transport via the Malaria Parasite’s Chloroquine Resistance Transporter. Science.

[53] Ecker A., Lehane A.M., Clain J., Fidock D.A. (2012). PfCRT and its role in antimalarial drug resistance. Trends Parasitol..

[54] Fidock D.A., Nomura T., Talley A.K., Cooper R.A., Dzekunov S.M., Ferdig M.T., Ursos L.M., Sidhu A.B., Naudé B., Deitsch K.W. (2000). Mutations in the P. falciparum digestive vacuole transmembrane protein PfCRT and evidence for their role in chloroquine resistance. Mol. Cell.

[55] Darghouth D., Koehl B., Madalinski G., Heilier J.-F., Bovee P., Xu Y., Olivier M.-F., Bartolucci P., Benkerrou M., Pissard S. (2011). Pathophysiology of sickle cell disease is mirrored by the red blood cell metabolome. Blood.

[56] Silva D.G.H., Junior E.B., de Almeida E.A., Bonini-Domingos C.R. (2013). Oxidative stress in sickle cell disease: An overview of erythrocyte redox metabolism and current antioxidant therapeutic strategies. Free Radic. Biol. Med..

[57] Voskou S., Aslan M., Fanis P., Phylactides M., Kleanthous M. (2015). Oxidative stress in β-thalassaemia and sickle cell disease. Redox Biol..

[58] Choudhuri S., Ghosh B. (2024). Computational approach for decoding malaria drug targets from single-cell transcriptomics and finding potential drug molecule. Sci. Rep..

[59] Gyamfi E., Baum J. (2025). Malaria parasite phenotypic heterogeneity and the power of single-cell technologies. Trends Parasitol..

